# Mammalian target of rapamycin and head and neck squamous cell carcinoma

**DOI:** 10.1186/1758-3284-3-22

**Published:** 2011-04-24

**Authors:** Yu-Min Liao, Charles Kim, Yun Yen

**Affiliations:** 1 Division of Hematology and Oncology, Department of Internal Medicine, China Medical University Hospital, Taichung, 404, Taiwan; 2 Department of Molecular Pharmacology, Beckman Research Institute, City of Hope Comprehensive Cancer, Duarte, CA 91010, USA

## Abstract

Head and neck squamous cell carcinoma (HNSCC), a significant cause of cancer deaths worldwide, has multiple stepwise malignant evolutions. Mammalian target of rapamycin (mTOR) plays a critical role in tumor development, invasion, metastasis and angiogenesis that impact local recurrence and survival. mTOR can also act as a biomarker for personalized adjuvant therapy. In *in vivo *and *in vitro *studies, mTOR inhibitor suppresses tumor growth and sensitizes HNSCC to radiation, cytotoxic agents and epidermoid growth factor receptor inhibitors. We have reviewed the pathogenesis of HNSCC, mTOR pathway, mTOR inhibitor and the role of mTOR in HNSCC.

## Review

Head and neck squamous cell carcinoma (HNSCC) is the sixth most common cancer worldwide and accounts for approximately 650,000 new diagnoses and 350,000 cancer deaths every year[1]. Smoking and alcohol are the most well known carcinogens of HNSCC[2]. In some areas of Asia, chewing betel quid, a psychoactive substance that always contains areca nut, betel leaf and calcium hydroxide, is a distinct risk factor that exerts a synergistic effect with smoking and alcohol consumption for oral and laryngeal cancer[3,4]. In addition, the continuation of smoking and alcohol consumption after initial diagnosis of HNSCC increases the risk for secondary primary cancer[5]. Human papillomavirus (HPV), predominantly type 16, infection inducing genomic instability is another mechanism for tumorigenesis in the oropharynx that is distinct from the role of smoking or alcohol[6].

Surgery and radiotherapy are the main modality of HNSCC treatment[7]. Chemotherapy, acting as a radio-sensitizer, increases survival in locally advanced disease[8,9]. To treat early disease, surgery is preferred. Radiotherapy is an alterative method for organ preservation for laryngeal cancer[10,11]. In unresectable settings, concurrent cisplatin chemoradiotherapy that provides better disease free survival and overall survival than radiotherapy alone is the standard of care[9]. Surgery-treated, advanced patients with high risk factors can also obtain benefit of local and regional control and progression free survival by adding concurrent chemotherapy to postoperative radiotherapy[12]. Overall, the incorporation of concurrent chemoradiotherapy to management of HNSCC absolutely increases survival rate by 6.5% at year-five[13]. Recently, cetuximab, an epidermal growth factor receptor-specific monoclonal antibody, plus radiation were shown to improve survival rate as compared to radiation treatment alone[14]. However, a retrospect study suggests the duration of progression free survival and overall survival is shorter in patient receiving cetuximab plus radiation than those with cisplatin plus radiation[13]. Multi-modality treatment or targeted therapy containing management does not significantly improve overall survival.

HNSCC has a complex mechanism of carcinogenesis that involves multiple genetic abnormalities, stepwise evolution and signaling pathway alternation[7,15-18]. Alternations of p53, p16 and cyclin D1 (CCND1) result in limitless growth of tumor cells[4,19-22]. Change of epidermal growth factor receptor (EGFR), c-MET, phosphatidylinositol 3-kinase, catalytic, alpha polypeptide (PIK3CA), Ras-mitogen-activate protein kinase (Ras-MAPK), phosphatase and tensin homolog (PTEN) and transforming growth factor-beta (TGF-beta) are essential to affect growth factor signaling that impact cell proliferation, apoptosis and survival[23-28]. High expression of nuclear factor Kappa B (NF-Kappa B), surviving and B cell lymphoma -2 (Bcl-2) are positively associated with poor survival[29-31].

### Target of rapamycin (TOR) pathway

**Figure 1:**
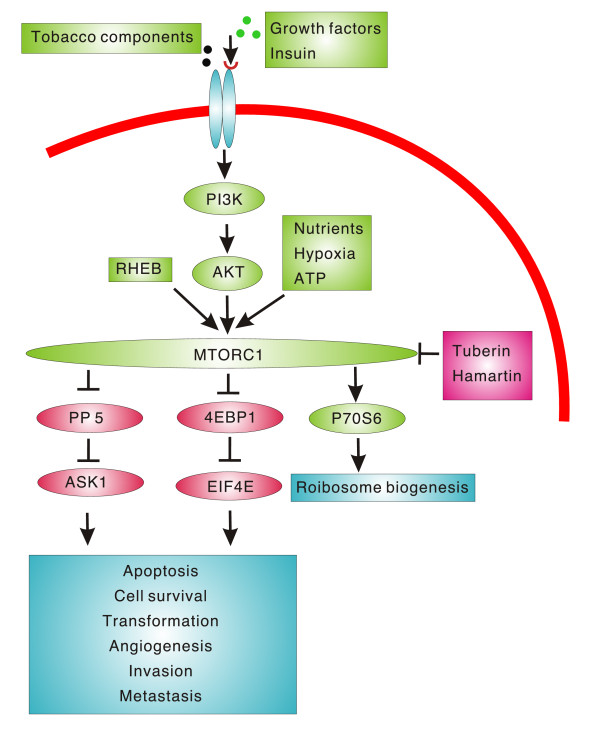
**Mammalian target of rapamycin is a key regulator in development and progression of cancer**. Mammalian target of rapamycin responds to stimuli of growth factor, insulin, tobacco components, nutrients, hypoxia, ATP and RHEB to activate P70S6 and inhibit 4EBP1 and PP5 with subsequent dysregulation of apoptosis, cell survival, cell transformation, tumorigenesis, angiogenesis, invasion and metastasis. PI3K, phosphatidylinositol 3-kinase; ATP, adenosine triphosphate; RHEB, ras homologue enriched in brain; mTORC1, mammalian target of rapamycin complex 1; PP5, protein phosphatase 5; ASK1, apoptosis-signal-regulating kinase 1; p70S6, ribosomal p70 S6; EIF4E, eukaryotic translation initiation factor 4E; 4E-BP1, EIF4E-binding protein 1.

Mammalian TOR (mTOR), a protein kinase encoded by FK506 binding protein 12-rapamycin associated protein 1 (FRAP1) gene[32]., is an important downstream target signal of PI3K pathway. (Figure 1) [33]. The protein contains an 12-kDa FK506-binding protein (FKBP12), rapamycin binding domain, Huntington Elongation Factor 3 PR65/ATOR (HEAT) motifs, FK506 binding protein 12-rapamycin associated protein (FRAP1)-ataxia telangiectasia mutated (ATM)-transformation transcription domain-associated protein (FAT) and FAT C terminus (FATC) domain. In terms of structure and function, mTOR consists of two distinct complexes: mTOR complex 1 (mTORC1) and mTOR complex 2 (mTORC2)[34,35]. mTOR, regulatory-associated protein of mTOR (Raptor) and G-protein-subunit-like protein form mTORC1, a nutrition-sensitive complex. mTORC1 is sensitive to rapamycin, control cell growth and is a key factor of the mTOR pathway[34-38]. mTORC2, a complex containing mTOR, G-protein-subunit-like protein and mAVO3, regulates the actin cytoskeleton and is insensitive to rapamycin[39]. As an important target kinase of the PI3K pathway, mTOR responds to multiple stimuli including: nutrients, insulin, oxygen, growth factor, ATP, Ras homologue enriched in brain (RHEB) and tobacco components[33,38,40-44]. However, mTOR is negatively regulated by complex of tuberin and hamartin[45]. Through the activation of two downstream targets p70S6K and 4EBP1, mTOR functions on translation, cell growth, protein synthesis, cell size and angiogenesis[46-48]. Activated p70S6K stimulates 5-terminal oligopyrimidine (5'TOG) translation to regulate ribosome biogenesis[49]. Phosphorylated 4EBP1 disassociates with eIF4E. The free eIF4E, an oncoprotein, promotes cap-dependent translation with subsequent regulation of c-myc, cyclin D1, ornithinedecarboxylase, basic fibroblast growth factor (b-FGF), vascular endothelial growth factor (VEGF) and matrix metalloproteinase-9 (MMP-9) to affect cell survival, tumorigenesis and transformation, angiogenesis, invasion and metastasis[41,50-54]. In addition, mTOR-enhanced expression of HIF-1a protein, HIF-1 transcriptional activity, and VEGF protein are the key regulators in angiogenesis[55]. Apoptosis signal-regulating kinase 1 (ASK1)-modulated apoptosis can be inhibited by mTOR-induced overexpression of protein phosphatase 5 (PP5)[56].

### mTOR inhibitor

Many compounds, including rapamycin, rapalog and adenosine-5'-triphosphate (ATP)-competitive inhibitor, have been shown to block the activation of the mTOR pathway[57]. Rapamycin, an antifungal agent,[58]. binds to the FKBP12-rapamycin (FRB) domain of mTORC1 to interrupt downstream activation[59]. Poor water solubility, absorption, limited bio-availability, hepatic first-pass effect and drug interaction account for interpatient variability that requires therapeutic drug monitoring for the complex pharmacokinetic behaviors[60]. Although rapamycin is a promisingly cytostatic anticancer agent in the National Cancer Institute's screening program,[35]. those pharmacologic characteristics limit the practical application[61,62]. In order to improve the pharmacokinetic features of rapamycin, a chemical modification at C-40-0 can develop three new rapalogs including everolimus, temsirolimus and ridaforolimus that share the same mechanisms of action. They not only exert anti-cancer activity but also act as a sensitizer to radiotherapy and chemotherapy. Frequent adverse events such as fatigue, mucositis, rash, anorexia, diarrhea, nausea, thrombocytopenia, leucopenia, anemia, hyperglycemia, hyperlipidemia and hypercholesterolemia are limited and manageable[63]. Everolimus (RAD001) is an oral rapalog, and has better oral absorption and bioavailability profiles than compared to those of rapamycin[64,65]. It also shows sustained inhibition of S6K1 activity at the dose of ≥ 20 mg weekly and ≥ 5 mg daily[66]. Temsirolimus is a prodrug converted into rapamycin after intravenous infusion. It exerts evidence of activity over a dose range between 15 and 300 mg/m^2^, and has the dose-limiting toxicity from thrombocytopenia[67]. Ridaforolimus is a non-prodrug rapalog, and has dose limiting toxicity from mouth sore at 28 mg/d and maximal tolerable dose of 18.5 mg/d[68]. In a study of skin biopsy specimens, ridaforolimus significantly suppressed the expression of 4EBP1, S6 and extracellular signal-regulated kinase (ERK)[69].

### mTOR pathway and HNSCC

HNSCC amplifies eukaryotic translation initiation factor 4E(eIF4E) gene and overexpresses eIF4E protein[70]. The tumor itself, the surgical margins, and even the histologically normal epithelium in the margins were all shown to overexpress eIF4E. The strong association of elevated activity of eIF4E with high expression of mTOR downstream signals transduction (phospho-4E binding protein 1, S6, phospho-mTOR) and elevated level of AKT expression suggests the activation of AKT/mTOR pathway in the margin. High expression of phospho-P70S6 and AKT in the margin indicates that the activity of AKT/mTOR cascade is higher in tumor margin than in the tumor itself[71]. There is a significant correlation between degree of expression of eIF4E in the margin and grading of the dysplasia (*P *= .006)[72]. eIF4E is essential in the malignant progression of HNSCC[70]. Interestingly, higher activity of eIF4E in the tumor margin, even those free of microscopic tumor, is an independent predictor of local recurrence while histological grading of dysplasia failed to predict prognosis[73]. Nathan et al examined tumor samples from 65 patients. All biopsies expressed elevated levels of eIF4E. The intra-tumor activity of eIF4E was not different between the eIF4E-positive and -negative margin groups. Thirty-six patients (55%) with microscopic tumor-free margins had eIF4E expression in the basal cell layer of the margin. After a median follow-up of 17 months, local-regional recurrence developed in 20 patients (56%) with eIF4E-positive margins. In contrast, two patients (6.9%) with absence of eIF4F expression had local recurrence after median follow-up of 14.5 months. Histologically tumor-free margin with high expression of eIF4E has a seven-fold risk of local failure. The median disease free duration is significantly shorter in the eIF4E positive margin group (eIF4E positive versus negative, 11 months versus 14 months; log-rank test, *P *= .0001). The prediction of recurrence by expression of eIF4E in HNSCC margin is independent from tumor size, nodal status, stage, histologic grade, tumor site, eIF4E levels in the tumor, and with the degree of dysplasia in the margins[72]. Also, the level of p-S6 expression significantly increases with the malignant progression of the tumor[74]. In addition, irradiation, an important treatment of HNSCC, promotes the expression of mTOR and AKT in HNSCC cells[75]. High expression of AKT sensitizes mTOR inhibition through down-regulation of cyclin D1 and c-myc[76,77]. Activation of AKT/mTOR pathway plays a key role in tumorigenesis and survival rate of HNSCC patients[71]. The eIF4E is a potential maker to define the molecular-free surgical margin, and is a promising predictor of survival[72,73].

### mTOR inhibitor and HNSCC

Temsirolimus blocks the activation of mTOR pathway in HNSCC cell line to reduce the expression of S6 and 4EBP1 with subsequently suppressed expression of FGF and VEGF that inhibited cell growth *in vitro*. In a xenograft study, the 4EBP1 activity of tumor cells and peripheral blood mononuclear cells (PBMC) is also reduced by mTOR inhibition[78,79]. Rapamycin treatment increased nuclei apoptosis in tumor *in situ *TUNEL assay, and reduced neovascularization[74]. To mimic patients with histologically tumor-free margin with high expression of eIF4E, the tumor cells in the culture medium were introduced into the dorsal flap of nude mice with pipettes to establish a minimal residual disease model (MRD). Measuring the tumor formation at day 21 after xenograft, the treatment group had a significantly longer median tumor free duration (treatment versus control group, 18 days versus 7 days; *P *< 0.0001). The tumor size of treatment group was significantly smaller than those of the control group (*P *< 0.0001). In the "survival study" mTOR inhibition delayed the time to develop tumors with the volumes of at least 200 m^3 ^in the MRD model (*P *< 0.0001). Twenty-one percent of the treated mice were free of tumors 30 days after the discontinuation of the treatment. As expected, temsirolimus treatment significantly reduced photon emission on bioluminescence imaging. The reduction increased with the continuation of the treatment. The result of the MRD model suggests that the prolonged mTOR inhibition may have clinical benefits in the adjuvant setting for patients with eIF4E positive margin[78]. mTOR inhibitor is a potential agent in HNSCC treatment. Phosphorylated mTOR, eIF4E, and high expression of AKT may be potentional biomarkers in order to select the candidate HNSCC patients for mTOR inhibitor-based adjuvant therapy[71,77,80].

Everolimus enhances DNA-damage agent-induced apoptosis in tumor cells. It overcomes cisplatin resistance in small cell and non-small cell lung cancer cell lines,[81,82]. and sensitizes cancer cells to radiation by arresting cells in G2M phase[79,83,84]. In an *in vivo *study, temsirolimus was shown to block signal transduction of mTOR pathway to decrease VEGF production, but failed to sensitize HNSCC to radiation by clonogenic assay. In a study with cisplatin-sensitive Fa-Du and cisplatin-resistant SCC-40 xerografts receiving 3-week treatment with temsirolimus or cisplatin plus radiation, temsirolimus alone treatment, even at low doses, significantly blocked the tumor growth in both xenografts. The combination of temsirolimus with radiation (XRT) more significantly promoted radiation-induced tumor reduction (*P < 0.05; *temsirolimus plus XRT versus. temsirolimus or XRT alone) than compared to the combination of cisplaint with XRT alone in both cisplaitn- sensitive and resistant cell lines (*P < 0.05*). Addition of cisplatin to the temsirolimus and XRT treatment failed to increase the therapeutic effect. The sensitization effect by temsirolimus is evidenced by the following: the reduced phosphorylation of 4EBP1, S6 and Bad; the increased number of radiation-related poly (ADP-ribose) polymerases (PARPs) cleavage; the increased rate of nuclei apoptosis; and the reduction of tumor vascularity by diminishing VEGF production. The median survival time was 49 days for the temsirolimus plus XRT treatment group, 38 days for the cisplatin plus XRT treatment group and 27 days for the control group for the SCC-40 cell lines. Treatment with temsirolimus alone or with the combination of XRT can significantly increase the survival rate of SCC-40 xenograft as compared to the control group (*P *< 0.05). mTOR inhibitor is a promising radio-sensitizer in HNSCC treatment[75]. Although EGFR is an important target of therapy,[85]. HNSCC poorly responds to or is refractory to anti-EGFR treatment. In HNSCC cell lines Detroit 562, erlotinib blocks the activation of MAPK and suppresses the expression of AKT and p70. Temsirolimus alone failed to affect AKT and MAPK. The MAPK was completely blocked by the combination treatment while the activity of AKT was significantly inhibited. In an *in vivo *study, the combination therapy, erlotinib alone therapy, and the temsirolimus alone therapy obtained growth rates that was 18%, 34% and 13% of the rate of growth of the control group, respectively. Seven days after the treatment, the expression of pMAPK, Ki-67 and phospho-p70 were significantly reduced. mTOR inhibition suppressed tumor growth of EGFR-resistant cell lines and exerted an additive effect with the combination of the EGFR inhibitor[86].

Few HNSCC patients were enrolled into a phase 1 study to investigate the safety of an mTOR inhibitor based combination therapy. A patient with HNSCC T4N3M1 with lung metastasis with failed responses to docetaxel, cisplatin and zalutumumab partially responded to temsirolimus and metformine[87]. One oropharyngeal cancer patient obtained stable disease after more than 6 cycles of treatments with everolimus and weekly cisplatin. No change of expression of p21, p53 or p-AKT was found on a biopsy specimen from pretreatment and day 21 on treatment[88].

Many studies have been initiated to elucidate the role of mTOR inhibitor in the treatment of HNSCC (Table 1). National Institutes of Dental and Craniofacial Research initiated a pilot study to investigate the efficacy and molecular change of neoadjuvant 3-week treatment of rapamycin in resectable HNSCC patients. Molecular study of the specimens obtained from tumor biopsies during the period of treatment provides further information for clinical response to rapamycin (clinicaltrial.gov identifier: NCT01195922). One future randamized phase II trial of everolimus versus placebo as an adjuvant therapy in patients with locally advanced HNSCC (NCT01111058) will evaluate the benefit of long-term mTOR inhibition in patients with eIF4E positive margin[78]. Some trials will test the safety at different dosages and determine the optimal dose of mTOR inhibitor in combination with radiation or cytotoxic agents.

**Table 1:** Clinical study for mTOR inhibitor in treatment of HNSCC

Drug	Study phase	Treatment design	Disease status	Clinicaltrial.gov identifier
Rapamycin	I/II	Neoadjuvant with 21-day rapamycin followed by surgery	Stage III or IVA, resectable	NCT01195922

Temsirolimus	II	Temsirolimus with or without cetuximab	Recurrent or metastasis	NCT01256385

	II	Temsirolimus alone	Recurrent or metastasis	NCT01172769

	I/II	Temsirolimus + Weekly paclitaxel + carboplatin	Recurrent or metastasis	NCT01016769

	I/II	Temsirolimus, cisplatin, and cetuximab	Recurrent or metastasis	NCT01015664

	II	Temsirolimus and erlotinib	Platinum-refractory or -ineligible, advanced disease	NCT01009203

Everolimus	I	Everolimus, weekly cisplatin and XRT	Locally advanced	NCT01058408

	I	Induction with everolimus, docetaxel, and cisplatin	Locally advanced	NCT00935961

	I	Everolimus, weekly cisplatin and XRT	Locally advanced	NCT00858663

	I	Everolimus, cisplatin and XRT	Locally advanced	NCT01057277

	I/II	Induction everolimus paclitaxel, and cisplatin	Locally advanced	NCT01133678

	II, randomized	Adjuvant everolimus Vs placebo	Locally advanced disease after definite local treatment	NCT01111058

	I/IIB	Everolimus, carboplatin, and cetuximab	Recurrent or metastaasis	NCT01283334

	I/II	Everolimus, cetuximab and cisplatin	Recurrent or metastaasis	NCT01009346

	II	Everolimus	Refractory, recurrent or metastasis	NCT01051791

	II	Everolimus, erlotinib	Recurrent	NCT00942734

Ridaforolimus	I	Ridaforolimus, cetuximab	Advanced	NCT01212627

## Conclusion

mTOR plays an important role in the complex carcinogenesis of HNSCC, predicts survival, and may be a potential biomaker to identify candidate patients for mTOR inhibition-based adjuvant therapy. Many preclinical experements suggest that the mTOR blockade has anti-tumor activity, displays radio- or chemo-sensitization, and overcomes the EGFR resistance. Further clinical trial results may provide more information about the role of mTOR in future studies and management of HNSCC.

## Competing interests

The authors declare that they have no competing interests.

## Authors' contributions

YY designed the paper. YY, YML and CK wrote the paper. All authors read and approved the final manuscript.
